# Scanning Electron Microscopic Comparison of Emblica officinalis Extract, EDTA, and Maleic Acid for Smear Layer Removal From Root Canal Dentin

**DOI:** 10.7759/cureus.111694

**Published:** 2026-06-28

**Authors:** Lakshmi Sunkara, Sunil Kumar Chennuru, Vamsee Krishna Nallagatla, Sadamsetty Sunil Kumar, Kalluru Subbarathnam Chandrababu, Ramisetty Bharathisuma

**Affiliations:** 1 Conservative Dentistry and Endodontics, Chadalawada Krishna Srinivasa (CKS) Teja Institute of Dental Sciences and Research, Tirupati, IND

**Keywords:** edta, emblica officinalis, maleic acid, root canal irrigation, scanning electron microscopy, smear layer

## Abstract

Background: Formation of a smear layer during canal preparation impedes the penetration of irrigants, medicaments, and sealers, posing a challenge to optimal endodontic results. This study compared the efficacy of 6.25% *Emblica officinalis *extract, 17% ethylenediaminetetraacetic acid (EDTA), 7% maleic acid, and normal saline in removing the smear layer from root canal dentin.

Methods: A total of 48 single-rooted human teeth extracted with fully developed apices were decoronated, standardized to 17 mm, and instrumented using a hybrid technique. Samples were randomly assigned to four groups of 12 (n = 12) as follows: group I - 17% EDTA, group II - 6.25% *Emblica officinalis* extract, group III - 7% maleic acid, and group IV - normal saline (control). Each canal was irrigated with 5 mL of the assigned solution for 1 min using a side-vented needle. Longitudinally sectioned specimens were observed under scanning electron microscopy (SEM), and the extent of smear layer elimination across the coronal, middle, and apical thirds was determined using Hülsmann’s criteria. Data were analyzed using one-way analysis of variance, Tukey's post hoc test, and Kruskal-Wallis analysis as a confirmatory test (p < 0.05).

Results: Seven percent maleic acid demonstrated significantly greater smear layer removal in the apical and middle thirds than the other irrigants (p < 0.05). Seventeen percent EDTA showed comparable efficacy in the coronal third. The 6.25% aqueous extract of *Emblica officinalis* exhibited moderate smear layer removal. Normal saline was the least effective irrigant across all canal regions.

Conclusions: Maleic acid at 7% emerged as the most effective irrigant for smear layer removal, surpassing 17% EDTA, 6.25% aqueous extract of *Emblica officinalis*, and normal saline. These results clearly indicate that conventional chelating agents, especially maleic acid, are superior to the evaluated herbal irrigant in achieving effective smear layer removal from root canal dentin.

## Introduction

Successful root canal therapy depends on effective cleaning and disinfection of the root canal system. This is achieved by removing microorganisms, necrotic tissue, and debris. Mechanical instrumentation is essential for canal shaping. However, it cannot completely debride the complex root canal anatomy, such as lateral canals, isthmuses, and apical ramifications. Therefore, chemical irrigation plays a crucial role in enhancing canal cleanliness, reducing microbial load, and facilitating smear layer removal [1].

The smear layer, first described by McComb and Smith, is generated during instrumentation and consists of microorganisms, necrotic tissue remnants, organic and inorganic debris, and odontoblastic processes [2]. This layer may penetrate dentinal tubules and provide a favorable environment for bacterial survival. Although the clinical significance of smear layer removal remains controversial, available evidence suggests that its removal improves sealer penetration, enhances obturation quality, and reduces microleakage, thereby contributing to the long-term success of endodontic treatment [3].

Root canal irrigation complements mechanical preparation by flushing out debris, dissolving tissue remnants, and assisting in the removal of the smear layer. The effectiveness of irrigation depends on several factors, including the type and concentration of irrigant, contact time, volume delivered, and method of application [4]. Sodium hypochlorite (NaOCl) is widely used for its potent antimicrobial and tissue-dissolving properties; however, it is ineffective at removing the inorganic component of the smear layer [5]. Consequently, chelating agents such as 17% ethylenediaminetetraacetic acid (EDTA) are routinely used to remove inorganic debris by chelating calcium ions [6]. Another chelating agent, 7% maleic acid, has demonstrated promising smear layer removal, particularly in the apical third, owing to its lower surface tension and superior penetration into dentinal tubules [7].

In recent years, increasing attention has been directed toward herbal irrigants because of their biocompatibility, antimicrobial activity, and reduced cytotoxicity [8]. *Emblica officinalis* (amla) contains several bioactive compounds, including ascorbic acid, tannins, and polyphenols, with antioxidant and antimicrobial properties. These constituents may also exhibit mild metal ion-binding (chelating) activity, which could contribute to root canal disinfection and partial smear layer removal [9]. Unlike conventional chemical irrigants, herbal alternatives may offer improved biological compatibility while maintaining acceptable cleaning efficacy. Normal saline is frequently used as a flushing solution during endodontic procedures. However, it lacks antimicrobial and tissue-dissolving properties, so it serves primarily as a control irrigant [10,11].

Despite the availability of various chemical and herbal irrigating solutions, no single irrigant possesses all the ideal characteristics required for effective root canal debridement. Limited studies have investigated the smear layer removal potential of *Emblica officinalis*, especially when contrasted with established agents such as EDTA and maleic acid. Using scanning electron microscopy (SEM) evaluation, the present in vitro investigation compared the smear layer removal potential of 17% EDTA, 7% maleic acid, 6.25% aqueous *Emblica officinalis* extract, and normal saline on root canal dentin using Hülsmann's scoring criteria in the coronal, middle, and apical thirds of instrumented root canals. The primary outcome measure was the smear layer score obtained at each canal level. The null hypothesis tested was that no statistically significant differences would exist among the four irrigating solutions with respect to smear layer removal in the evaluated regions of the root canal.

## Materials and methods

This in vitro study was approved by the Institutional Ethics Committee of Chadalawada Krishna Srinivasa (CKS) Teja Institute of Dental Sciences and Research, Tirupati, India (#CKS/ENDO/23-24/03). The study sample comprised 48 extracted human single-rooted teeth, selected based on sample sizes reported in previously published in vitro studies evaluating smear layer removal using scanning electron microscopy. As no preliminary data were available to estimate the effect size for a formal power analysis, a convenience sample comparable to those used in similar investigations was employed to ensure adequate intergroup comparison while maintaining methodological consistency [12]. Teeth with fully developed roots, closed apices, intact external surfaces, and normal anatomical morphology were included. Teeth with open apices, previous endodontic treatment, root caries, internal resorption, calcified canals, root fractures, cracks, or other pathological alterations were excluded to ensure specimen uniformity and minimize confounding factors.

Following collection, adherent soft tissue remnants were removed, and the specimens were decontaminated according to standard sterilization protocols. The crowns were sectioned using a diamond disc under water cooling, and all root specimens were standardized to a length of 17 mm. Access cavities were prepared, and biomechanical preparation was performed using a hybrid instrumentation technique. Canal enlargement was completed to a maximum of 3 file sizes beyond the initial apical file using stainless-steel K-files. Coronal flaring was performed with Gates-Glidden drills up to size 3 to facilitate irrigant penetration. During instrumentation, 2.5% sodium hypochlorite was used between successive files for disinfection and debris removal. After completion of canal preparation, all specimens received a final rinse with 5 mL of normal saline.

The prepared specimens were randomly assigned to four experimental groups (n = 12) using a computer-generated randomization sequence. Group I received 17% EDTA. Group II received 6.25% aqueous extract of *Emblica officinalis* (amla). Group III received 7% maleic acid. Group IV received normal saline, which served as the control. A 27-gauge side-vented needle positioned 2 mm short of the working length was used to deliver 5 mL of the respective irrigant for 1 min. Following this, canals were washed with distilled water and dried using absorbent paper points.

**Figure 1:**
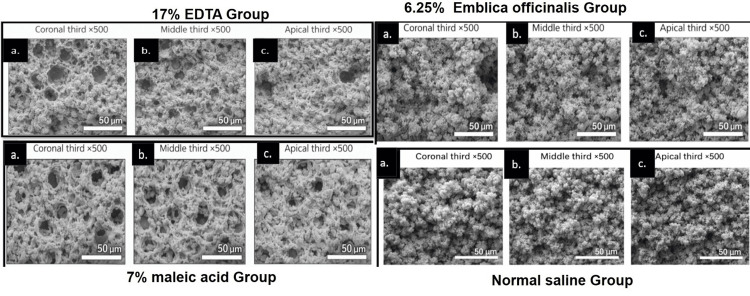
Representative SEM images (×500) demonstrating smear layer removal following irrigation with 17% EDTA (A), 6.25% aqueous extract of Emblica officinalis (B), 7% maleic acid (C), and normal saline (D). SEM: scanning electron microscopy; EDTA: ethylenediaminetetraacetic acid

**Figure 2:**
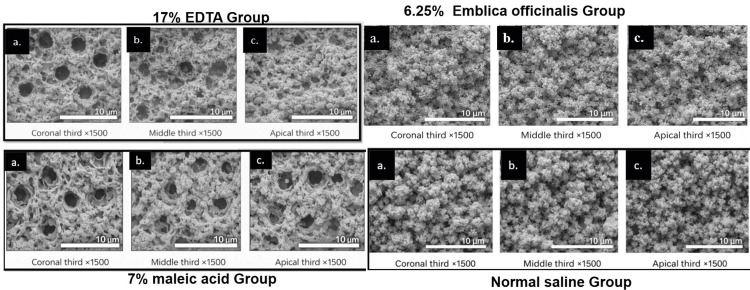
Representative SEM images (×1500) demonstrating smear layer removal following irrigation with 17% EDTA (A), 6.25% aqueous extract of Emblica officinalis (B), 7% maleic acid (C), and normal saline (D). Maleic acid and EDTA exhibited greater dentinal tubule patency, whereas normal saline demonstrated a dense smear layer covering the canal walls. SEM: scanning electron microscopy; EDTA: ethylenediaminetetraacetic acid

Longitudinal grooves were created along the buccal and lingual aspects of each root for scanning electron microscopy (SEM) analysis, ensuring they did not invade the root canal space. Each root was subsequently divided into two halves by applying controlled force with a chisel and mallet. To prevent debris generated during sectioning from contaminating the canal walls, absorbent paper points were placed in the canals before splitting. One-half of each specimen was selected for SEM analysis. The specimens were examined at magnifications of ×500 and ×1500, and digital micrographs were obtained from the coronal, middle, and apical thirds of the root canal (Figures 1, 2).

Smear layer removal was scored according to the study by Hülsmann et al. - score 1, no smear layer, all tubules open; score 2, little smear layer, most tubules open; score 3, uniform smear layer, few tubules visible; score 4, complete wall coverage, no tubules visible; and score 5, heavy, nonuniform smear layer covering the canal wall [13]. The coronal, middle, and apical thirds were evaluated independently.

Statistical analysis was conducted using SPSS version 23.0 (Armonk, NY: IBM Corp.). Data were summarized as mean ± standard deviation. Intergroup comparisons were performed using one-way ANOVA followed by Tukey's post hoc test. A Kruskal-Wallis test was also employed as a confirmatory analysis because the smear layer scores were ordinal. Statistical significance was established at p < 0.05.

## Results

The mean smear layer scores in the apical, middle, and coronal thirds among the experimental groups are presented in Table 1. Lower smear layer scores indicated superior cleaning efficacy. In the apical third, 7% maleic acid demonstrated the lowest mean smear layer score (2.4 ± 0.52), followed by 17% EDTA (2.8 ± 0.42), 6.25% aqueous extract of *Emblica officinalis* (3.4 ± 0.52), and normal saline (4.5 ± 0.53). Similar findings were observed in the middle third, where 7% maleic acid exhibited the greatest smear layer removal (1.8 ± 0.42), followed by 17% EDTA (2.3 ± 0.48), amla extract (2.8 ± 0.42), and normal saline (4.3 ± 0.48). In the coronal third, 17% EDTA showed the lowest mean smear layer score (1.3 ± 0.48), closely followed by 7% maleic acid (1.8 ± 0.42). Amla extract demonstrated moderate efficacy, whereas normal saline consistently exhibited the highest smear layer scores in all thirds, indicating the least cleaning ability (Table 1).

**Table 1:** Descriptive statistics of smear layer scores among the experimental groups. Values are presented as mean ± standard deviation. EDTA: ethylenediaminetetraacetic acid

Irrigants	n	Apical (mean ± SD)	Middle (mean ± SD)	Coronal (mean ± SD)	95% CI
7% maleic acid	12	2.4 ± 0.52	1.8 ± 0.42	1.8 ± 0.42	2.07-2.73
17% EDTA	12	2.8 ± 0.42	2.3 ± 0.48	1.3 ± 0.48	2.53-3.07
6.25% amla extract	12	3.4 ± 0.52	2.8 ± 0.42	2.8 ± 0.42	3.07-3.73
Normal saline	12	4.5 ± 0.53	4.3 ± 0.48	4.3 ± 0.48	4.16-4.84

One-way analysis of variance (ANOVA) revealed statistically significant differences in smear layer removal among the experimental groups in all root canal regions. In the apical third, a significant intergroup difference was observed (F = 11.82, p = 0.001). Likewise, highly significant differences were noted in the middle (F = 14.67, p < 0.001) and coronal thirds (F = 14.67, p < 0.001), suggesting that the type of irrigant significantly influenced smear layer removal (Table 2).

**Table 2:** Intergroup comparison of smear layer scores using one-way ANOVA. *P < 0.05 was statistically significant.

Root canal third	n	Sum of the squares	df	Mean square	F-values	p-Values
Apical	12	22.60	3	7.53	11.82	0.001*
Middle	12	28.75	3	9.58	14.67	<0.001*
Coronal	12	28.75	3	9.58	14.67	<0.001*

Post hoc pairwise comparisons using Tukey's test demonstrated that 7% maleic acid significantly removed the smear layer more than amla extract and normal saline across all thirds of the canal. In the apical third, maleic acid performed significantly better than EDTA (p = 0.041). Similar findings were observed in the middle third (p = 0.038). In the coronal third, no statistically significant difference was observed between EDTA and maleic acid (p = 0.052), indicating comparable cleaning efficacy in this region (Table 3).

**Table 3:** Pairwise comparisons using Tukey's post hoc test. *P < 0.05 was statistically significant. EDTA: ethylenediaminetetraacetic acid

Comparison	Apical p-Value	Middle p-Value	Coronal p-Value
Maleic acid vs. EDTA	0.041*	0.038*	0.052
Maleic acid vs. amla extract	0.001*	0.001*	0.001*
Maleic acid vs. normal saline	<0.001*	<0.001*	<0.001*
EDTA vs. amla extract	0.032*	0.044*	<0.001*
EDTA vs. normal saline	<0.001*	<0.001*	<0.001*
Amla extract vs. normal saline	0.002*	0.001*	0*

To substantiate the findings from the parametric analysis, a Kruskal-Wallis test was performed as a confirmatory nonparametric analysis. Significant differences among the experimental groups were observed in the apical (χ² = 18.62, df = 3, p < 0.001), middle (χ² = 20.14, df = 3, p < 0.001), and coronal thirds (χ² = 22.35, df = 3, p < 0.001). These findings corroborated the ANOVA results, confirming that the type of irrigant significantly influenced smear layer removal from root canal dentin (Table 4).

**Table 4:** Kruskal-Wallis test as a confirmatory analysis. *P < 0.05 was statistically significant.

Root canal third	χ² value	df	p-Value
Apical	18.62	3	<0.001*
Middle	20.14	3	<0.001*
Coronal	22.35	3	<0.001*

## Discussion

Successful root canal therapy depends on effective biomechanical preparation and chemical irrigation to eliminate microorganisms, necrotic tissue, and debris while facilitating optimal obturation [4]. The smear layer generated during instrumentation consists of dentinal debris, remnants of pulp tissue, odontoblastic processes, microorganisms, and their by-products, which may hinder the penetration of irrigants, intracanal medicaments, and sealers into dentinal tubules [5,14,15]. In addition, the smear layer can serve as a reservoir for residual bacteria, interfere with the adaptation and bonding of obturation materials, increase the risk of apical and coronal microleakage, and compromise the long-term success of endodontic treatment. Therefore, effective removal of the smear layer remains an important objective in endodontic therapy to facilitate canal disinfection, improve sealer penetration and adhesion, and enhance the quality and longevity of the root canal seal.

The present in vitro study compared the smear layer removal efficacy of 17% EDTA, 7% maleic acid, 6.25% aqueous extract of *Emblica officinalis* (amla), and normal saline using SEM evaluation. The findings demonstrated that 7% maleic acid exhibited superior smear layer removal, particularly in the apical and middle thirds of the canal, whereas 17% EDTA showed comparable efficacy in the coronal third. The 6.25% aqueous amla extract demonstrated moderate cleaning efficacy, while normal saline was the least effective irrigant across all regions.

The superior smear layer removal achieved with maleic acid in the present study is consistent with previous investigations reporting enhanced cleaning efficacy, particularly in the apical third of root canals, which has been attributed to its low surface tension, acidic nature, and improved wettability that facilitate deeper penetration into narrow canal spaces and dentinal tubules [16-18]. Ballal et al. demonstrated that 7% maleic acid was more effective than 17% EDTA in removing the smear layer, especially in the apical third of instrumented canals [19]. The enhanced efficacy of maleic acid may also be related to its smaller molecular size and greater affinity for calcium ions within hydroxyapatite, enabling efficient dissolution of the inorganic component of the smear layer. Conversely, some studies have reported comparable smear layer removal between maleic acid and EDTA, suggesting that factors such as irrigation time, delivery techniques, contact duration, irrigant activation, and canal morphology may influence their relative performance [17,20]. Although evidence from in vitro SEM studies consistently supports the smear layer removal potential of maleic acid, direct clinical evidence remains limited because assessment of smear layer removal largely relies on scanning electron microscopic evaluation of extracted teeth. Nevertheless, available evidence suggests that maleic acid is a promising final irrigant because of its effective demineralizing action, low surface tension, and improved dentin wettability, which may facilitate better sealer adaptation and penetration. However, most supporting evidence is derived from in vitro investigations, highlighting the need for well-designed clinical studies to validate its routine use [17].

EDTA remains the most extensively investigated chelating agent in endodontics and is considered the gold standard for smear layer removal when used in conjunction with sodium hypochlorite [15,21]. Nygaard-Østby first introduced EDTA into endodontic practice because of its ability to chelate calcium ions and decalcify dentin, thereby facilitating canal instrumentation and smear layer removal [22]. Subsequent studies by Goldman et al. demonstrated that a final rinse with 17% EDTA effectively removes the inorganic component of the smear layer, exposing dentinal tubules and enhancing the adaptation and penetration of sealers [23]. Similar findings were reported by Çalt and Serper, who observed that 17% EDTA applied for 1 min efficiently eliminated the smear layer, whereas prolonged exposure led to undesirable dentinal erosion, emphasizing the importance of controlled application [24].

In the present study, 17% EDTA was effective in removing the smear layer, particularly in the coronal and middle thirds, although its efficacy was reduced in the apical third compared with that of 7% maleic acid. These findings corroborate those of Ballal et al., who reported superior smear layer removal with maleic acid in the apical region, attributing the difference to its lower surface tension and enhanced penetration into constricted canal spaces [19]. Recent evidence suggests that the relatively neutral pH, larger molecular size, and self-limiting chelating mechanism of EDTA may reduce its decalcification potential in sclerosed apical dentin, thereby limiting its effectiveness in the apical third [6,20]. Although several studies have demonstrated superior apical smear layer removal with maleic acid, the overall effectiveness of chelating agents may be influenced by factors such as irrigant contact time, canal anatomy, delivery systems, and activation techniques, resulting in comparable outcomes under certain experimental conditions [3].

In recent years, herbal irrigants have attracted increasing attention in endodontics due to their biocompatibility, antioxidant activity, antimicrobial potential, and reduced cytotoxicity compared with conventional chemical irrigants. Various plant-derived irrigants, including *Azadirachta indica* (neem), *Camellia sinensis* (green tea), *Curcuma longa* (turmeric), *Morinda citrifolia* (noni), *Aloe barbadensis* (aloe vera), *Ocimum sanctum* (tulsi), and *Emblica officinalis* (amla), have been investigated as alternative or adjunctive endodontic irrigants. Their therapeutic properties are primarily attributed to the presence of polyphenols, flavonoids, tannins, alkaloids, and organic acids, which exhibit antimicrobial, anti-inflammatory, and antioxidant effects [8,25,26].

Among these herbal agents, *Emblica officinalis* has attracted attention due to its high levels of ascorbic acid, gallic acid, ellagic acid, emblicanin A and B, and hydrolyzable tannins. These bioactive constituents may contribute to mild chelating activity, inhibition of microbial growth, and neutralization of reactive oxygen species. The use of a 6.25% aqueous extract in the present study was based on previous investigations demonstrating acceptable antimicrobial efficacy and favorable biocompatibility at this concentration while minimizing the risk of dentinal alterations associated with stronger acidic solutions [9]. Despite exhibiting significantly better smear layer removal than normal saline, amla extract was less effective than EDTA and maleic acid, which may be explained by its comparatively weaker chelating ability, higher viscosity, and reduced capacity to penetrate deeply into dentinal tubules. Nevertheless, its biological advantages suggest that amla may serve as a potential adjunctive irrigant, particularly in situations where enhanced biocompatibility and reduced cytotoxicity are desired.

SEM analysis remains a reliable and widely accepted method for evaluating smear layer removal because it provides high-resolution visualization of canal walls, residual debris, and dentinal tubule patency [17]. The present findings further emphasize that effective smear layer removal depends not only on the chemical properties of irrigants but also on factors such as irrigant delivery systems, surface tension, instrumentation techniques, and activation protocols. Previous studies have shown that sonic and ultrasonic agitation significantly enhance irrigant penetration and smear layer removal, particularly in the apical third of the canal [20,27]. In the present study, the use of side-vented needles enabled controlled delivery of irrigant while minimizing the risk of apical extrusion, thereby simulating clinically acceptable irrigation procedures. Standardization of canal preparation and irrigation protocols is essential to minimize methodological variability and permit reliable comparison of smear layer removal among different irrigants [5,19].

From a clinical perspective, the findings of this study support the use of a combined irrigation strategy involving sodium hypochlorite for dissolution of organic tissue and a chelating agent for elimination of the inorganic component of the smear layer. Maleic acid may be considered a promising alternative to EDTA, particularly for improving cleanliness in the apical third, whereas herbal agents such as amla may serve as adjunctive irrigants when enhanced biocompatibility is desirable. Selection of an appropriate irrigation regimen based on physicochemical properties, efficacy, and biological safety may improve smear layer removal, enhance sealer penetration, and ultimately contribute to favorable long-term endodontic outcomes.

The present study was limited by its in vitro design, relatively small sample size, and evaluation of only a single concentration of each irrigant. In addition, only immediate smear layer removal was assessed, while other clinically relevant outcomes, such as antimicrobial efficacy, dentinal erosion, microhardness, bond strength, and long-term alterations in dentin properties, were not investigated. Physicochemical characterization of the 6.25% *Emblica officinalis* extract, including pH, surface tension, and phytochemical standardization, as well as inter-examiner reliability assessment for SEM analysis, was not performed. Future in vivo studies with larger sample sizes should evaluate different irrigant concentrations, activation techniques, and combinations of conventional and herbal agents to establish clinically relevant irrigation protocols.

## Conclusions

Among the irrigants evaluated, 7% maleic acid exhibited the greatest smear layer removal efficacy, likely due to its low surface tension, acidic nature, and enhanced penetration into dentinal tubules. Seventeen percent EDTA also demonstrated effective smear layer removal because of its established calcium-chelating properties. The 6.25% aqueous extract of *Emblica officinalis* (amla) showed moderate efficacy, suggesting its potential as a biocompatible adjunct irrigant, whereas normal saline exhibited minimal smear layer removal. Overall, conventional chelating agents, particularly 7% maleic acid and 17% EDTA, were more effective in achieving thorough smear layer removal than the herbal irrigant evaluated in this study.
